# The Effect of Acupuncture to SP6 on Skin Temperature Changes of SP6 and SP10: An Observation of “Deqi”

**DOI:** 10.1155/2014/595963

**Published:** 2014-02-10

**Authors:** Jia-Min Yang, Xiao-Yu Shen, Ling Zhang, Song-Xi Shen, Dan-Dan Qi, Shi-Peng Zhu, Li Luo, Xiao-Xuan Ren, Bo Ji, Lu-Fen Zhang, Xiao-Hong Li, Jiang Zhu

**Affiliations:** School of Acupuncture, Moxibustion and Tuina, Beijing University of Chinese Medicine, No. 11 Beisanhuan Donglu, Chaoyang District, Beijing 100029, China

## Abstract

*Background*. Deqi sensation is a complex but an important component for acupuncture effect. In this study, we tried to observe the relationship between Deqi and skin temperature changes and whether there was some relativity between Deqi and needle stimulations on cold congealing and dysmenorrhea rat model. Thirty-two female Sprague Dawley (SD) rats were randomly divided into four groups (Saline Control Group, Model Group, Group A with strong stimulation, and Group B with small stimulation). Group A and Group B were performed with different stimulations. We found that, compared with saline control group, model group, and Group B, Group A showed that the skin temperature changes on right acupoint SP6 and SP10 increased significantly at 5 min–10 min interval. The skin temperature changes on left SP6 decreased at instant–5 min interval. The skin temperature changes on right SP10 decreased significantly at instant–5 min interval and 10 min–20 min interval. Thermogenic action along Spleen Meridian of Foot Greater Yin was manifested as simultaneous skin temperature increase on right SP6 and SP10 at 5 min–10 min interval after needling SP6, which was helpful to illustrate the relationship between the characteristic of Deqi and needle stimulations.

## 1. Introduction

Acupuncture is an important component of traditional Chinese medicine (TCM) for at least 3000 years. The meridian and collateral theories of TCM are considered to be the guideline for treating disease by acupuncture. The vital energy (essence) and qi are circulating along meridian and collaterals. Acupoints, not isolated, are special points on the surface of the body where the vital energy (qi and blood) of the viscera infuses. Therefore, internal disease may be reflected on acupoints through meridians. Needling-specific acupoints can treat the corresponding internal disease. Therefore acupoints are often utilized as important locations for disease diagnosis and treatment [1].

Deqi which literally means “obtaining qi” serves as an important component in the process of achieving therapeutic effect in acupuncture treatment. Deqi is a kind of meridian sensation after acupuncture which includes needling sensation felt by both the patient, and the practitioner [2]. The needle sensation refers to the localized sensation from the patient, such as soreness, numbness, heaviness, and distension—that may transmit to other places along the course of meridians. The practitioner will feel mild tightness around the needle. Deqi is also used as a sign for therapeutic effect and judging whether the acupuncture manipulation is appropriate. There is a close relationship between Deqi and the therapeutic effect, just as the statement in *Neijing* (*Inner Classic of Yellow Emperor*) refers to the excitation of qi inside meridians by means of needle stimulation. In Chapter 1 of *Ling Shu* (*Miraculous Pivot*), it states, “For acupuncture to be successful, the qi must be arrived (qi arrival or obtaining qi). Acupuncture's effects come about like the clouds blown away by the wind.” In *Biao You Fu*, it says, “Therapeutic effectiveness come with quick qi arrival, and ineffectiveness present without qi arrival.” This indicates that Deqi has direct relationship with therapeutic efficiency and Deqi can also be used to measure the practitioner's technique. Needle manipulation, direction, and depth of needle insertion can be used to promote needle sensation. Although many basic and clinical researches regarded needle sensation such as soreness, numbness, heaviness, distension, and pain as the indication of Deqi, it is noteworthy that it does not mean needle sensation can be used as the unique standard for Deqi and clinical therapeutic effect.

According to traditional acupuncture theory, the main factors which affect Deqi include body state, acupoints, needles, and needle manipulation. Acupoints are not only closely related to corresponding meridian, collateral, and viscera organs, they can also be utilized to regulate the function of visceral organs. When human body is in a morbid state, acupoint becomes much more sensitive and the sensitized acupoint will manifest as acupoint specificity under the external stimulations—namely “small stimulation, strong reaction” [3]. Needling a certain acupoint can induce various affects away from the needled acupoint [4].

Related literature reports that Deqi sensation is transmitted by different nerve fibers. Aching, soreness, distention, heaviness, warmth, and dull pain are conveyed by A*δ* and C fibers (slower conducting fibers), whereas numbness is conveyed by A*β* fibers (faster conducting fibers). The distribution of nerve has close relationship with tissue structures. For instance, A*δ* and C fibers are more intensively distributed on the tendon layer. So needling on the tendon layer may induce more sensations of aching, soreness, distention, and warmth [5]. Related research demonstrated that stabbing pain and sharp pain were more obvious on the shallow layer while dull, heaviness, radiating, and electric sensation were much easier to be induced on the deeper layer than the shallow layer, which elucidated that the deeper layer could excite more Deqi sensation [6]. Based on these researches, length of the needle and the insertion depth were considered as stimulation factors.

Needle manipulation is usually used to induce or strengthen needle sensation (Deqi). Acupuncture can influence the skin temperature, just as *Ling Shu (Miraculous Pivot)* says that the doctor could warm the body through stimulating foot Shaoyin channel and cool the body through stimulating foot Yangming channel [7]. In order to explore the relationship between Deqi and skin temperature changes, and whether there is direct relativity between Deqi and needle stimulus intensity. In this study, we punctured acupoint SP6 by different needle stimulations and observed the skin temperature changes of acupoint SP6 and SP10 along meridians by applying infrared thermal camera.

## 2. Material and Methods

### 2.1. Animal

Thirty-two female Sprague-Dawley (SD) rats aged 3 months, weighing 220 g ± 10 g, matured and unmated (which were purchased from the Academy of Military Medical Science, Beijing, China). All rats were caged individually and kept in a constant environment with food and water freely available throughout the experiment. The rooms were maintained at 23°C ± 1°C with humidity 45% ± 5% and a standard 12-hour light-and-dark cycle (lights on at 08:00 am). Testing was performed during the light cycle. Animals were allowed to habituate in the environment for one week before experiment. The principles of laboratory animal care (NIH publication No. 86-23, revised 1985) were followed in the experiments.

### 2.2. Establishment of Cold Congealing and Dysmenorrhea Rat Model

*Step 1.* Female SD rats undergoing diestrus were selected through vagina smear. The selected rats were subcutaneously injected Estradiol Benzoate for 0.5 mg/rat on the 1st and 10th day, and 0.2 mg/rat from the 2nd to the 9th day.

*Step 2.* Then, the rats were placed in a freezer for 4 hours at the first 5 days with temperature maintained at −25°C and the freezer was opened for 5 seconds during 2-hour interval. The rats can be removed from the freezer when the rats manifested as shivering, aversion to cold and preference for warmth, curled up and falling asleep, dull reaction, feeble respiration, fluffy and lusterless fur, light-colored urine, loose stool, dark reddish ear, and purplish nail and tail.

*Step 3.* 0.2 unit Oxytocin was injected intraperitoneally 1 hour later after the injection of Estradiol Benzoate on the 10th day. Saline Control Group was given the same saline dosage for ten days.

### 2.3. Grouping

Thirty-two female Sprague-Dawley (SD) rats were randomly divided into four groups by using a random number table, Saline Control Group (*n* = 8), Model Group (*n* = 8), Group A (“Deqi” group) (*n* = 8), and Group B (No “Deqi” group) (*n* = 8).

### 2.4. Needling Method

**Figure 1:**
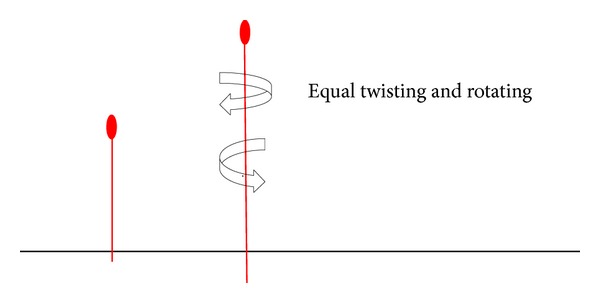
Equal twisting and rotating acupuncture needle manipulation.

Acupuncture stimulation was done manually using disposable acupuncture needles (Zhongyan Taihe brand, Wuxi, China). Sanyinjiao (SP6) is located 10 mm proximal to the prominence of the medial malleolus. SP6 were needled bilaterally in Group A (“Deqi” group, cold congealing and dysmenorrhea model with Needle Stimulation A) and Group B (No “Deqi” group, cold congealing and dysmenorrhea model with Needle Stimulation B). Needle Stimulation A was performed with diameter 0.25 mm × length 25 mm filiform needle and perpendicularly needled for 4 mm-5 mm with equal reinforcing and reducing manipulation (evenly twirling and rotating for 30 seconds at instant needling and 10 min later after needling). Needle Stimulation B was performed with diameter 0.22 mm × length 13 mm filiform needle and subcutaneously needled without needle manipulation. Needles were retained for 20 min. Model Group and Saline Control Group were performed without needling. (See Figure 1.)

### 2.5. Infrared Thermal Imaging of Animal

**Figure 2:**
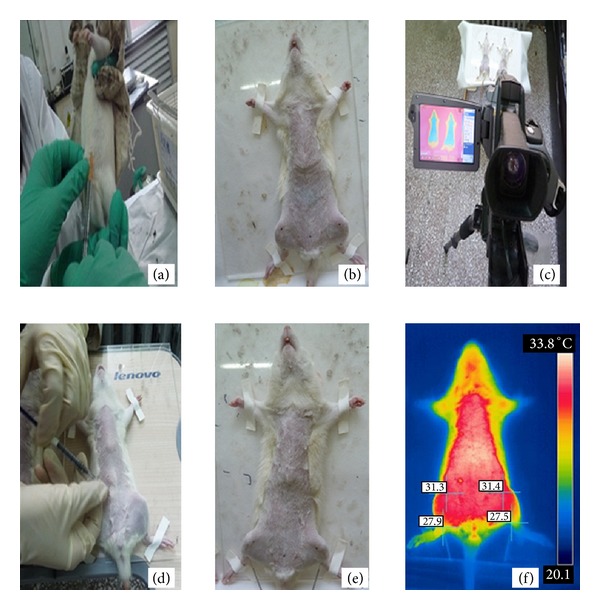
(a) Subcutaneous injection 0.5 mg Estradiol Benzoate, (b) Mark SP6 and SP10, (c) record the baseline temperature, (d) subcutaneous injection 2-unit oxytocin, (e) needling SP6, and (f) take images at instant, 5 min, 10 min, 20 min, 30 min, 40 min, 50 min, and 60 min after needling.

**Figure 3:**
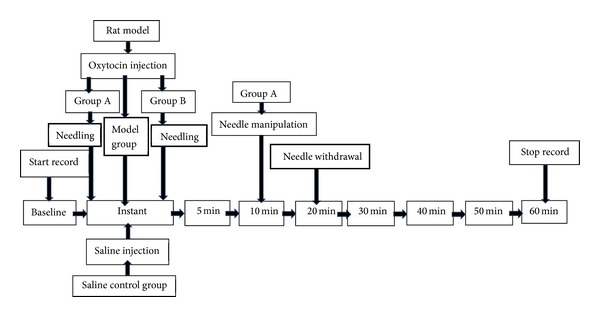
Acupuncture procedure and infrared thermal imaging.

Infrared thermal camera (FLIR SC640, FLIR system, America), with wide temperature range from −40°F to 3632°F (−40°C to 2000°C), FOV 25°  ×  19°, and thermal sensitivity of 0.1°C was used in this study. At the tenth day of establishing cold congealing and dysmenorrhea rat model, 0.5 mg Estradiol Benzoate was subcutaneously injected at the lower abdomen and 0.7 mL/100 g Urethane was given by intramuscular injection for anesthesia. One hour later, the front fur was shaved by an electric hair clipper. Rats were fixed on a transplant board afterwards. Thermal images were captured with the indoor temperature and humidity maintained homogenously at 23°C ± 1°C and 60% ± 5%. The anterior aspect of the rat was placed perpendicularly from the infrared thermal camera with a constant distance of 1.0 meter, and then both SP6 and SP10 were marked on the computer. Record the basal skin temperature of SP6 and SP10. Then, 2-unit Oxytocin was given intraperitoneal injection. Both left and right SP6 were needled in Group A and Group B whereas Saline Control Group and Model Group without needling. Record the skin temperature of SP6 and SP10 at baseline without needling, instant, 5 min, 10 min, 20 min (withdrawal the needle), 30 min, 40 min, 50 min, and 60 min after needling. The experiment procedure is as follows (Figures 2 and 3).

### 2.6. Statistical Analysis

SAS 9.3 software was used for statistical analysis. Excel was used for graph generation. Skin temperature changes on SP6 and SP10 in the four groups were calculated as different time intervals (such as baseline-instant, instant–5 min, 5 min–10 min, 10 min–20 min, 20 min–30 min, 30 min–40 min, 40 min–50 min, and 50 min–60 min). The data were analyzed with one-way ANOVA or Nonparametric Test. LSD's test was used for comparing the skin temperature changes between different groups. Skin temperature changes were compared at different time intervals among Saline Control Group, Model Group, Group A, and Group B. The data was presented as Mean ± SD. The statistical significance was set at *P* < 0.05. −*P*_1_ stands for the significant difference between Group A and Saline Control Group; −*P*_2_ stands for the significant difference between Group A and Model Group; −*P*_3_ stands for the significant difference between Group A and Group B; and −*P* value stands for the significant difference between Group A and Saline Control Group, and Model Group, and Group B.

## 3. Result

### 3.1. Effect of Skin Temperature Changes on Left SP6 at Different Time Intervals

**Figure 4:**
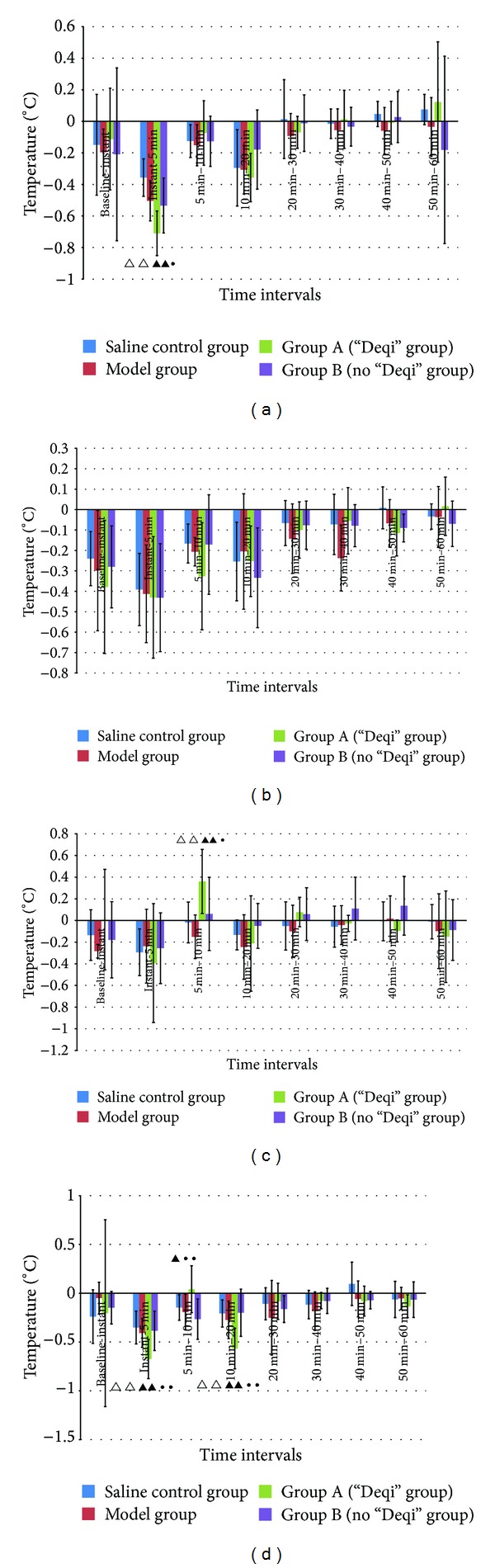
(a) Bar chart shows that skin temperature changes on left SP6 (Mean ± SD, *n* = 8). *X*-axis indicates different time intervals such as baseline–instant interval, instant–5 min interval, 5 min–10 min interval, 10 min–20 min interval, 20 min–30 min interval, 30 min–40 min interval, 40 min–50 min interval, and 50 min–60 min interval. Note: △△ compared with Saline Control Group, *P*_1_ < 0.01. ▲▲ compared with Model Group, *P*_2_ < 0.01. • compared with Group B (No “Deqi” group), *P*_3_ < 0.05. (b) Bar chart shows that skin temperature changes on left SP10 (Mean ± SD, *n* = 8). *X*-axis indicates different time intervals the same as (a). (c) Bar chart shows that skin temperature changes on right SP6 (Mean ± SD, *n* = 8). *X*-axis indicates different time intervals the same as (a). Note: △△ compared with Saline Control Group, *P*_1_ < 0.01. ▲▲ compared with Model Group, *P*_2_ < 0.01. • compared with Group B (No “Deqi” group), *P*_3_ < 0.05. (d) Bar chart shows that skin temperature changes on right SP10 (Mean ± SD, *n* = 8). *X*-axis indicates different time intervals the same as (a). Note: △△ compared with Saline Control Group, *P*_1_ < 0.01. ▲▲ compared with Model Group, *P*_2_ < 0.01. ▲ compared with Model Group, *P*_2_ < 0.05. •• compared with Group B (No “Deqi” group), *P*_3_ < 0.01.

Skin temperature changes on left SP6 in Group A significantly decreased at instant–5 min interval as compared to that in Saline Control Group (*P*_1_ = 0.000 < 0.01), Model Group (*P*_2_ = 0.007 < 0.01), and Group B (*P*_3_ = 0.019 < 0.05). See Figure 4(a).

### 3.2. Effect of Skin Temperature Changes on Left SP10 at Different Time Intervals

There was no significant difference at different time intervals among Saline Control Group, Model Group, Group A, and Group B as *P* value >0.05. See Figure 4(b).

### 3.3. Effect of Skin Temperature Changes on Right SP6 at Different Time Intervals

In Group A, skin temperature changes on right SP6 significantly increased at 5 min–10 min interval as compared to that in Saline Control Group (*P*_1_ = 0.008 < 0.01), Model Group (*P*_2_ = 0.001 < 0.01), and Group B (*P*_3_ = 0.031 < 0.05). See Figure 4(c).

### 3.4. Effect of Skin Temperature Changes on Right SP10 at Different Time Intervals

Skin temperature changes on right SP10 in Group A decreased significantly at instant needling–5 min interval as compared to that in Saline Control Group (*P*_1_ = 0.001 < 0.01), Model Group (*P*_2_ = 0.006 < 0.01), and Group B (*P*_3_ = 0.003 < 0.01). See Figure 4(d).

Skin temperature changes on right SP10 in Group A increased prominently at 5 min–10 min interval as compared to that in Saline Control Group (*P*_1_ = 0.063 > 0.05), Model Group (*P*_2_ = 0.023 < 0.05), and Group B (*P*_3_ = 0.004 < 0.01). See Figure 4(d).

Skin temperature changes on right SP10 in Group A decreased prominently at 10 min–20 min interval as compared to that in Saline Control Group (*P*_1_ = 0.001 < 0.01), Model Group (*P*_2_ = 0.006 < 0.01), and Group B (*P*_3_ = 0.001 < 0.01). See Figure 4(d).

## 4. Discussion

The aim of the present study was to observe the relationship between Deqi and skin temperature changes and whether there was some relativity between Deqi and needle stimulations. This study showed that there was simultaneous skin temperature increasing effect on right SP6 and SP10 in Group A, whereas there was no such effect on SP6 and SP10 in Group B. Thus, we found that the simultaneous skin temperature changes along meridian could be considered as Deqi phenomena. There is some relativity between Deqi and needle stimulation, which is that Deqi phenomena could be induced easier by strong stimulation than small one.

Lü et al. [8] discovered that high temperature belt which appeared after needling had a close relationship with the intensity of needle sensation. More distinct high temperature belt would appear when higher intensity of needle sensation was induced. The high temperature belt appeared along the circulating pathway of meridians only when Deqi (obtaining qi) arrived. In this study, Figure 4(d) indicated that Needle Stimulation A (thicker needle with deep insertion and equal reinforcing and reducing manipulation) could induce the skin temperature increase effect along meridians (Deqi), while stimulation B (thinner needle with subcutaneously insertion without equal reinforcing and reducing manipulation) could not induce temperature increase effect along meridians. This result provided evidences for the relativity between Deqi and needle stimulus intensity.

Previous clinical investigation showed that skin temperature on the acupoints region increased when patients felt a kind of heat sensation passing along meridians after needling some acupoints. The skin around the needled acupoints often turned to be red and hot which indicated that needling acupoint can induce skin temperature changes. The results in Figure 4(d) showed that skin temperature changes decreased prominently at instant–5 min interval and 10 min–20 min interval in Group A (“Deqi” group). Just as the previous literature reported, Lü et al. [8] discovered that there was a temporary reaction after needling acupoints instantly, manifested as dark thermal images which indicated the skin temperature decreased. The skin around the needled acupoints was flushed and skin temperature on the needled area increased soon afterwards. The area where skin temperature increased was much wider than the flushed area. High temperature belts which appeared in the thermal images were in accordance with traditional acupuncture meridians. A German subject who did not know meridian and collateral theories said that needle sensation (similarly like a worm crawling) circulated along the lateral side of the forearm after needling Yangchi (SJ4). Based on the previous literature, infrared thermal imaging technique was applied to the experimental research on acupuncture Deqi.

Infrared thermal imaging technique is a nontouching, audio-visual, accurate, sensitive, quick, safe, and widely used technology to detect the surface temperature distribution. Based on the photographing mechanism of infrared thermal imaging, superficial temperature of the subject can be reflected directly. The accuracy of infrared thermal imaging technique was affected by emissivity, reflectivity (or absorptivity), environment temperature and radiation of the high temperature object nearby [9]. The superficial heat of bodies can be dissipated through heat radiation, evaporation, heat conduction, air movement, and so forth. In the basic windless environment, heat dissipation caused by air movement is very little. While there is a ratio as listed below—heat radiation/evaporation/heat conduction = 1/0.3/0.1 [10]. The influence of heat dissipation through evaporation, heat conduction, and air movement is so little that it can be ignored. In this study, the environment temperature and humidity were maintained at 23°C ± 1°C and 60% ± 5%, respectively. So temperature on the thermal images can reflect the skin temperature of the body.

Many literature reported that acupuncture could regulate autonomic nerve activities by decreasing the activities of sympathetic function and manifested as vasodilatation, blood flow increase, and skin temperature increase, and so forth [11]. Results in Figures 4(b) and 4(d) showed the effect of simultaneous skin temperature increased at 5 min–10 min interval in Group A (“Deqi” group), which may have some connection with the acupuncture regulating effect. The simultaneous skin temperature increased on SP6 and SP10 only manifested on the right side which showed that there may be different sensitivity to skin temperature changes between left and right meridians.

Acupoint is the location where meridian qi infuses. It can regulate the dysfunction of related internal organs through dredging the channels and promoting qi to activate blood after needling. So the disease of internal organs can be reflected on corresponding meridians and acupoints. When the human body is diseased, acupoints on the body surface may have various types of sensitization. Acupoint heat-sensitization is a type of such sensitization. The sensitized acupoints showed acupoint-specific “small stimulation inducing large response” by external relative stimulation [3].

Based on the previous study on primary dysmenorrhea (PD) and Sanyinjiao (SP6), SP6 was the most effective and commonly used acupoint to cure PD [12]. According to the correlation theory between meridian points and Zang-fu organs [13], SP6 may become more sensitive to PD. Thus, in this study, we established cold congealing and dysmenorrhea rat model to observe Deqi, the skin temperature changes along spleen meridians by needling Acupoint SP6. Cold congealing and dysmenorrhea rat model was established by combining both cold congealing model and dysmenorrhea model together. Syndrome of cold congealing model was established by freezing the rat in a −25°C freezer. Dysmenorrhea rat model was established by subcutaneous injection of Estradiol Benzoate (which can improve the Oxytocin sensitivity to smooth muscle) and intraperitoneal injection of Oxytocin (which can make the smooth muscle contract) [14–17].

Acupoint SP6 pertains to spleen meridian of foot greater yin and is a crossing point of three yin meridians of foot (such as spleen meridian, liver meridian, and kidney meridian). So, SP6 has the function of regulating meridian qi of the liver, spleen, and kidney, tonifying spleen earth, promoting transportation and transformation, dredging qi stagnation, regulating blood and the uterus, and removing wind dampness from the meridian and collaterals. SP6 has a close relationship with the uterus, the thoroughfare vessel (sea of blood), conception vessel (sea of yin meridians); and governor vessel (sea of yang meridians), all the vessels connect together to regulate yin, yang, qi, and blood of the uterus. SP10 (Xuehai) is located on spleen channel of foot greater yin. It has the function of reinforcing qi and controlling blood, cooling blood and extinguishing wind, invigorating spleen and resolving dampness, detoxing and relieving itching, dredging channels and activating collaterals, removing stasis, and relieving pain. SP6 and SP10 are commonly selected to cure primary dysmenorrhea (PD) in clinic [12]. So, the phenomena that the simultaneous skin temperature increased on SP6 and SP10 area along spleen meridian after needling SP6 was utilized to elucidate the characteristic of Deqi.

## 5. Conclusion

The present findings indicate that there is direct relativity between Deqi and needle stimulus intensity. Different stimulation can induce different skin temperature changes on acupoints and exhibit different effect. The phenomena of skin temperature change along meridians can be used to illustrate Deqi of acupuncture. Most previous Deqi researches were performed on human beings; thus, Deqi phenomena (skin temperature changes along meridian) in this rat experiment study provide more evidence for animal experiment on acupuncture study and will be helpful for the further research on the therapeutic effect between Deqi and dysmenorrhea.
